# Conservative Management of Bowel Endometriosis: Cross-Sectional Analysis for Assessing Clinical Outcomes and Quality-of-Life

**DOI:** 10.3390/jcm13216574

**Published:** 2024-11-01

**Authors:** Marcello Ceccaroni, Silvia Baggio, Tommaso Capezzuoli, Mara Albanese, Paride Mainardi, Carlotta Zorzi, Giovanni Foti, Fabio Barra

**Affiliations:** 1 Department of Obstetrics and Gynecology, Gynecology Oncology and Minimally-Invasive Pelvic Surgery, International School of Surgical Anatomy (ISSA), IRCCS Ospedale Sacro Cuore—Don Calabria, Via Don A. Sempreboni 5, Negrar, 37024 Verona, Italy; 2 Department of Radiology, IRCCS Ospedale Sacro Cuore Don Calabria, Via Don A. Sempreboni 5, 37024 Negrar, Italy; 3 Unit of Obstetrics and Gynecology, P.O. “Ospedale del Tigullio”-ASL4, Via G. B. Ghio 9, Metropolitan Area of Genoa, 16043 Chiavari, Italy; fabio.barra@icloud.com; 4 Department of Health Sciences (DISSAL), University of Genoa, Via Antonio Pastore 1, 16132 Genova, Italy

**Keywords:** bowel endometriosis, surgery, medical treatment, conservative management, quality of life

## Abstract

**Background/Objectives**: Bowel endometriosis (BE) is characterized by the presence of endometrial-like tissue within the muscular layer of the bowel wall. When BE does not result in the severe obstruction to fecal transit and in the absence of (sub)occlusive symptoms, the decision to perform surgery can be challenging, as intestinal procedures are associated with higher complication rates and long-term bowel dysfunction. This cross-sectional study aims to evaluate the quality of life (QoL) in patients with BE who avoided surgery, as well as to investigate the impact of the characteristics of BE nodules on the QoL and intestinal function. **Methods**: A retrospective cross-sectional analysis was conducted involving 580 patients with BE who did not undergo surgery but were treated conservatively with medical therapy or expectant management between January 2017 and August 2022. The diagnosis of BE was established through transvaginal ultrasound and confirmed via double contrast barium enema. After at least one year of follow-up, the QoL and intestinal function were assessed using the Endometriosis Health Profile-5 (EHP-5) questionnaire and the Bowel Endometriosis Symptom (BENS) score, while pain symptoms were quantified with the Visual Analog Scale (VAS 0–10). Statistical analyses were performed to explore potential associations between the QoL and the characteristics of BE nodules (size, location, and evidence of stenosis), as well as the type and duration of medical therapy. **Results**: Patients with BE reported a satisfactory overall QoL, with a mean EHP-5 score of 105.42 ± 99.98 points and a VAS score below three across all pain domains. They did not demonstrate significant impairment in bowel function, as indicated by a mean BENS score of 4.89 ± 5.28 points. Notably, patients receiving medical therapy exhibited a better QoL compared to those not receiving treatment (*p* < 0.05), with the exception of postmenopausal patients, who reported the highest QoL overall (*p* < 0.05). Among the characteristics of BE, nodule location significantly impacted the QoL and symptom intensity, with low (rectal or rectosigmoid) nodules less tolerated compared to sigmoid nodules, particularly regarding non-menstrual pelvic pain (NMPP), dyschezia, and psychological impact on daily life (*p* < 0.05). **Conclusions**: Women can effectively manage BE conservatively in the absence of (sub)occlusive symptoms, even when large nodules are present, causing significant radiological stenosis. The characteristics of BE nodules do not significantly affect the QoL or symptom intensity; however, the location of BE nodules is a crucial factor negatively influencing these outcomes. Medical therapy may confer a beneficial impact on patients of reproductive age with BE, but its use should be carefully considered for those approaching menopause, weighing the risks and benefits.

## 1. Introduction

Bowel is the extragenital site most frequently affected by endometriosis. Bowel endometriosis (BE) is defined as the presence of endometrial-like glands not only in the serosa and subserosal tissue but also in the muscular layer of the bowel wall [1,2]. BE lesions are found in 8–12% of women with endometriosis, with 90% localized in the rectosigmoid region [1]. Typical symptoms of BE include abdominal bloating, diarrhea, and constipation; however, the disease can also be asymptomatic [3].

Treatment for BE remains a subject of debate: when endometriotic lesions cause bowel obstruction or severe sub-occlusion, surgery is necessary [4]. In other cases, both surgical and medical treatments can be suitable options. Some authors advocate for excisional surgery, arguing that medical treatments provide limited benefit, targeting the endometrial and smooth muscle components of the nodule but not the fibrotic tissue [5,6,7]. However, significant improvements in bowel symptoms have been observed during treatment with oral estrogen–progestin contraceptive pills (OCs) or progestin pills (Ps), thereby avoiding surgery [8]. Specifically, it was reported that more than 50% of women with symptomatic BE avoided surgery by using OCs or progestins [9]. In this context, the levonorgestrel-releasing intrauterine device (LNG-IUD) and contraceptive vaginal ring have proved to be effective therapeutic approaches for symptoms related to BE [10,11].

It is well known that the symptoms experienced by patients with endometriosis do not necessarily correlate with the disease burden [12]. Severe pelvic pain, chronicity of the disease, side effects of treatment, and lack of understanding by others can significantly impact the personal, psychological, and social aspects of patients’ daily lives [13]. Given that the decision to operate or to choose conservative treatment is primarily based on patient symptoms, it is crucial to utilize valid tools to assess symptom severity and quality of life (QoL), such as the Endometriosis Health Profile-30 (EHP-30) [14] and its shorter validated version, the Endometriosis Health Profile-5 (EHP-5) [15]. Although the EHP-30 and EHP-5 are very useful for evaluating the psychological and social aspects of endometriosis, they do not combine self-reported QoL with organ-specific symptoms. Therefore, other authors have developed a score to identify women with Bowel Endometriosis Syndrome (BENS) and to monitor the effects of medical and surgical management in women suffering from BE [16].

While the detrimental effects of BE on patients’ well-being [3,17] and the impact (both positive and negative) of bowel surgery are well documented [18], data on potential BE characteristics influencing QoL are limited. We hypothesize that specific BE characteristics, such as lesion location and size, and anamnestic factors, including the use of medical therapy in women of reproductive age and menopausal status, may significantly influence QoL. Therefore, the primary aim of this cross-sectional analysis of a retrospective cohort is to evaluate how these factors affect QoL and pain symptoms in conservatively treated patients who have successfully avoided surgical intervention.

## 2. Material and Methods

### 2.1. Study Population and Objectives

This is a cross-sectional analysis of a cohort of patients with a diagnosis of BE, conducted at the Department of Obstetrics and Gynecology, Gynecologic Oncology, and Minimally Invasive Pelvic Surgery, International School of Surgical Anatomy, IRCCS “Sacro Cuore-Don Calabria” Hospital in Negrar di Valpolicella, Verona (Italy), between January 2017 and August 2022. All subjects provided their informed consent before participating in this study. This study was conducted in accordance with the Declaration of Helsinki, and the protocol was approved by the Ethics Committee (Comitato Etico delle Province di Verona e Rovigo—secondary analysis of Prot. ULTRAPARAMETRENDO I and II—Prog. 3705CESC 9 March 2022).

The primary objective of this study is to evaluate the effects of BE characteristics (such as nodule location, size, degree of stenosis, and number of affected sites) and anamnestic factors (intake of medical therapy in women of reproductive age and menopausal status) on the QoL and pain symptoms of conservatively treated patients with BE who have avoided surgical intervention.

The diagnosis of BE was confirmed by both transvaginal sonography (TVS) and double-contrast barium enema (DCBE). Patients were included in this study if they had been treated conservatively with medical therapy or expectant management and had at least one year of follow-up from the diagnosis of BE. Exclusion criteria included a history of colorectal surgery (except for appendectomy), a previous surgical or radiological diagnosis of BE, previous bilateral ovariectomy, or psychiatric disorders.

After collecting clinical data and ultrasonographic characteristics of the patients at diagnosis, their current clinical situation and menopausal status were assessed through a phone call, during which pain was evaluated using a visual analog scale (VAS), and QoL was assessed through standardized questionnaires.

### 2.2. Imaging Methods

Ultrasound scans were performed on the same day as the DCBE by examiners with more than 10 years of experience in diagnosing endometriosis, using ultrasound machines (Samsung WS80A Elite, Via Mike Bongiorno. Milan, Lombardy, Italy) equipped with a wideband 5- to 9 MHz transducer. Detailed sonographic reports were created, and representative digital images of each patient were saved and stored on a hard drive for subsequent review and analysis. The ultrasound was performed using a transvaginal approach; for virgin patients, a transrectal approach was used, both integrated with transabdominal ultrasonography. The ultrasound was performed at any phase of the menstrual cycle, regardless of hormonal therapy. The examination was always conducted using a systematic, “step-by-step” approach, describing the sonographic features using terms, definitions, and measurements reported by the IDEA consensus [19].

**Figure 1:**
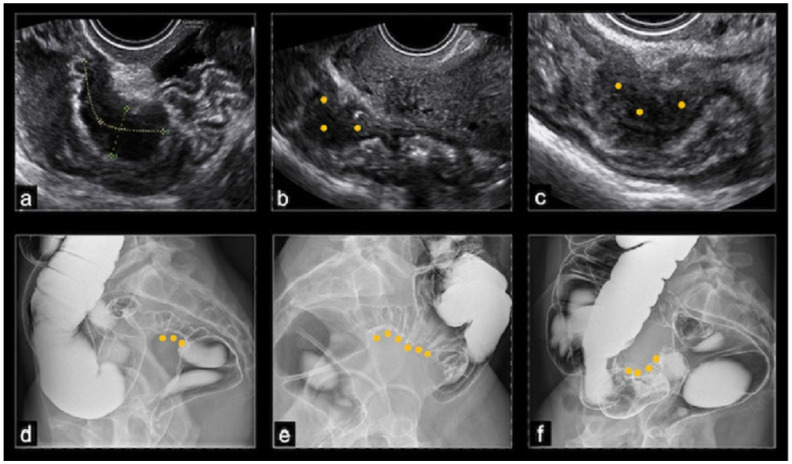
(**a**–**c**) Ultrasonographic images of rectal nodules causing an estimated stenosis of 30–40%; (**d**) barium enema image of a recto-sigmoid junction nodule causing an estimated stenosis of 30–50%; (**e**) barium enema image of a sigmoid nodule causing an estimated stenosis greater than 50%; (**f**) barium enema image showing a cecal nodule.

On ultrasound, BE typically appeared as a thickening of the hypoechoic muscularis propria or as hypoechoic nodules with irregular margins, without detectable blood flow on color Doppler. Nodules located above the level of the uterine fundus were considered sigmoid lesions, those at the level of the uterine fundus were denoted as rectosigmoid junction lesions, and those below this level were classified as rectal lesions. The dimensions of the nodules were recorded in three orthogonal planes; however, in this study, only the largest measurement was considered. The protrusion of a nodule toward the lumen of the bowel indicated the possibility of (sub)stenosis. The final nodule dimension was determined by averaging the dimensions reported by TVS and the DCBE, while the degree of stenosis was evaluated solely by the DCBE (Figure 1).

All DCBE procedures were performed using a Sireskop SX 40 fluoroscopy system with a motorized table tilt (Siemens AG Medical Engineering, Forchheim, Germany), equipped with the Fluorospot T.O.P. imaging system (Siemens AG Medical Engineering, Forchheim, Germany). The DCBE was not scheduled during a specific phase of the menstrual cycle. Patients were instructed to follow a low-residue diet for three days before the examination. On the day before the procedure, patients were given 13 oral tablets of Pursennid (glycosides of senna; Novartis Farma, Naples, Italy) and 15 g of magnesium sulfate, followed by 2 L of liquids to minimize dehydration caused by the preparation. In patients suspected of having rectal or rectosigmoid junction endometriosis, a 100% weight-to-volume ratio barium (Prontobario Colon; E-Z-EM, Inc., Westbury, NY, USA) was instilled into the rectum while the patient lay in the left lateral position. A first lateral view of the rectum was obtained. Once the barium reached the hepatic flexure, the colon was drained by gravity to empty the rectal ampulla of barium, without completely clearing the entire rectosigmoid colon. An anticholinergic agent, hyoscine N-butylbromide, was then used to induce colonic hypotonia. Room air was gently and intermittently insufflated into the colon. Each colonic segment was viewed in detail using spot radiographs and mid- to high-magnification digital images. Multiple spot films were obtained in all cases, and full-size films of the abdomen (overhead films) were taken in all cases. The procedure lasted an average of 15 min. The images were reviewed by a skilled gastrointestinal radiologist, who evaluated any extrinsic mass effect, kinking, shortening, or flattening of the bowel as indirect signs of endometriosis nodules. The size of the nodules and the degree of stenosis were calculated using specific radiologic software (Version 1). The final report included the number of BE nodules, their locations (rectum, rectosigmoid junction, sigmoid, ileum, cecum, or appendix), dimensions of the nodules, and degree of stenosis. Ileocecal and ileal BE detected with the DCBE were only considered in the descriptive part of this study and not included in the statistical analysis due to the high risk of intestinal obstruction associated with these types of lesions, where treatment was almost always surgical. A conservative approach was only evaluated in cases of small (less than 2 cm) and asymptomatic nodules.

### 2.3. Clinical Symptoms

Pain was assessed using a VAS for dysmenorrhea, non-menstrual pelvic pain (NMPP), dyspareunia, dysuria, and dyschezia. A 10-point scale was used, with 1 indicating “no pain” and 10 indicating “the worst imaginable pain”.

QoL was assessed for all patients using two validated instruments: the BENS score and the EHP-5 score.

The BENS score is the first endometriosis classification system that is based directly on the patient’s symptoms, investigating pelvic pain and QoL, as well as urinary, sexual, and bowel dysfunction. This tool has previously been shown to be useful in monitoring women with conservatively treated BE. The BENS score is obtained by summing the scores associated with each answer in the 7-item questionnaire. The range of the BENS score (0–28) is divided into three categories: 0–8 (no BENS), 9–16 (minor BENS), and 17–28 (major BENS) [16].

The EHP-5 is a questionnaire developed from the longer, validated EHP-30 to provide a brief instrument for measuring health outcomes for women with endometriosis. The EHP-5 is divided into two parts, with questions referring to the previous 4 weeks: a 5-item core questionnaire about pain, control, powerlessness, emotions, social support, and self-image, and an additional 6-item modular questionnaire about work life, relationships with children, sexual intercourse, medical profession, treatment, and infertility. The response system consists of five levels, ranked in order of severity: ‘never’ = 0, ‘rarely’ = 25, ‘sometimes’ = 50, ‘often’ = 75, and ‘always’ = 100. The modular questionnaire was developed to include items that may not be applicable to every woman with endometriosis. In this study, we chose to consider only the core part of the questionnaire to focus specifically on pain and to reduce the total interview time. The core part was shown to be effective in detecting pain intensity compared to other questionnaires [20]. The responses from the 5-item EHP-5 were summed and transformed according to the EHP-5 manual on a scale from 100 (worst possible QoL) to 0 (best possible QoL).

### 2.4. Statistical Analysis

Statistical analyses were conducted using SPSS for Windows, version 22.0 (SPSS Inc., Chicago, IL, USA).

The Kolmogorov–Smirnov test was utilized to assess the normality of data distribution, which informed the selection of statistical tests. For normally distributed data, the mean and standard deviation (SD) were reported, while non-normally distributed data were expressed as median and interquartile range (IQR). The t-test and ANOVA allowed for robust comparisons of quality of life (QoL) and symptom scores across groups with differing reproductive statuses and treatments, while the Kruskal–Wallis test was used for the non-normally distributed scores of QoL and bowel symptoms across the varying BE nodule characteristics (i.e., size, location, and stenosis). Statistical significance was defined as *p* < 0.05. Bonferroni adjustments were applied to control for type I error, ensuring that multiple comparisons did not inflate false-positive rates.

The selection of these tests ensured robust and reliable comparisons across patient groups and provided insights into how both medical therapy and BE characteristics impact pain, QoL, and bowel function.

## 3. Results

### 3.1. Demographic Characteristics

**Figure 2:**
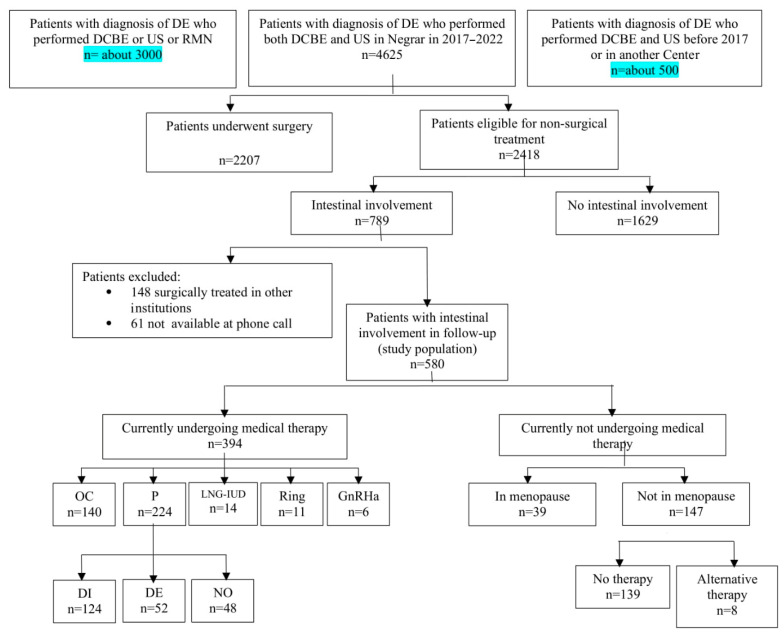
Flowchart of study population (DCBE: double contrast bowel enema; OC: combined oral contraceptives; P: progestins; LNG-IUD: levonorgestrel intrauterine device; GnRHa: Gonadotropin releasing hormone agonists; DI: dienogest; DE: desorgestrel; NO: norethisterone acetate; tp: therapy).

During the study period, 4625 women underwent the DCBE and TVS as second-level examinations for suspected deep endometriosis. Of these, 2207 (47.7%) had surgical indications, and surgery was performed in 47.9% of these cases. The remaining 2418 women (52.3%) were considered suitable for conservative, non-surgical treatment, with 789 (32.6%) of these cases involving suspected intestinal involvement. A flowchart of all patients is shown in Figure 2.

Following the follow-up phone call, it was discovered that 148 out of 789 patients (18.7%) had undergone surgery for deep endometriosis at other institutions and were thus excluded from this study. An additional 61 patients (7.7%) did not respond to the phone call and were therefore excluded (Figure 1).

The final analysis was conducted on 580 patients who had not undergone any gynecological surgery after being diagnosed with BE through imaging. Their characteristics are reported in Table 1. Among these patients, 302 (52.1%) had no prior history of surgery for endometriosis, 210 (36.2%) had undergone one previous surgery, and 68 (11.7%) had two or more surgeries for endometriosis. In most cases (95.3%), the prior surgeries were not radical and were often performed for diagnostic purposes or involved intraperitoneal procedures, such as the enucleation of endometriotic cysts. Only 9 patients (3.2%) had a previous bowel resection for deep endometriosis.

Three hundred ninety-four patients (67.9%) were still undergoing hormonal therapy, while 185 (32.1%) were not taking any medical therapy. Of these 185 women, 147 (79.5%) had residual ovarian activity with regular or irregular menses, while 39 (20.5%) were in menopause. Among patients undergoing therapy, 140 (35.5%) were taking OCs, with a continuous regimen in 86.4% of cases, either without a pause between blisters or with a 7-day pause every 4 months. All 224 women (56.8%) on progestin therapy followed a continuous regimen without interruption.

### 3.2. Quality of Life, Bowel Function, and Pain

The patients included in this study demonstrated a satisfactory overall QoL, with a mean (±SD) EHP-5 score of 105.42 ± 99.98 points, and good intestinal function, reflected by a mean BENS score of 4.89 ± 5.28. The mean VAS scores for dysmenorrhea and non-menstrual pelvic pain (NMPP) were 1.49 ± 2.72 and 2.74 ± 2.23, respectively.

In line with our hypothesis, we investigated the impact of two primary factors on pain symptoms, quality of life (QoL), and bowel function: (1) the characteristics of bowel endometriosis (BE), and (2) anamnestic factors.

### 3.3. Impact of Anamnestic Factors

A sub-analysis was conducted across three groups of patients: (a) those of reproductive age under medical therapy, (b) those of reproductive age not undergoing medical therapy, and (c) those in menopause (not undergoing medical therapy). The patients of reproductive age not undergoing medical treatment had medical or hematologic contraindications or intolerance to therapy. Table 2 reports the scores from the QoL questionnaires for the entire population and for these three groups of patients.

Patients of reproductive age undergoing medical therapy demonstrated better QoL compared to those not undergoing therapy, particularly in terms of intestinal symptoms: BENS (*p* = 0.001), pain (*p* = 0.001), control and powerlessness subdomains (EHP-5, *p* = 0.048), VAS scores for dysmenorrhea (*p* < 0.001), dyschezia (*p* < 0.001), dysuria (*p* = 0.032), and NMPP (*p* = 0.002).

Patients of reproductive age undergoing medical therapy showed worse QoL compared to those in menopause not receiving medical therapy in terms of total EHP-5 score (*p* < 0.001), intestinal symptoms on BENS (*p* = 0.06), VAS scores for dyschezia (*p* = 0.008) and NMPP (*p* = 0.009), as well as the control and powerlessness (*p* = 0.012), social support (*p* = 0.003), and self-image (*p* = 0.003) subdomains of the EHP-5.

When comparing OCs and progestins in patients undergoing medical therapy, all QoL scores (BENS, EHP-5, and VAS scores) were similar, except for the VAS score for dysmenorrhea, which was significantly better in patients receiving progestins (0.03 ± 0.34 vs. 0.83 ± 2.05 points). There was no inter-group difference based on the specific progestin used (dienogest, desogestrel, or norethisterone acetate; *p* > 0.05).

All these findings support our hypothesis by highlighting that hormonal and reproductive status play a critical role in symptom management and QoL, with menopausal women generally experiencing less pain and better psychosocial outcomes.

### 3.4. Impact of BE Nodule Characteristics

A last comparison evaluated the impact of BE characteristics (nodule size, location, and stenosis) on symptom intensity, as well as the relationship between the presence and duration of medical therapy and symptom severity.

The localization of BE influenced QoL: specifically, sigmoid nodules had a lower impact on QoL compared to rectosigmoid nodules in terms of VAS scores for dyschezia (*p* = 0.045), NMPP (*p* = 0.007), and the control and powerlessness subdomain of the EHP-5 (*p* = 0.019). Additionally, the VAS score for NMPP was lower in patients with sigmoid nodules compared to those with rectal nodules (*p* = 0.045). Patients with nodules larger than 3 cm had lower mean VAS scores for dysmenorrhea (*p* = 0.17) and lower BENS scores (*p* = 0.021) compared to those with smaller BE nodules. Notably, lower BENS scores (*p* = 0.019), better emotional well-being subdomain scores on the EHP-5 (*p* = 0.042), and lower VAS scores for dysmenorrhea (*p* = 0.028) and dyschezia (*p* = 0.047) were observed in patients with stenosis >30%. Otherwise, there were no differences in these scores between patients with no stenosis and those with stenosis between 20% and 30%.

Among the 72 patients with symptoms that had a significant impact on QoL (BENS score ≥ 9 and EHP-5 total score ≥ 200), 41 (56.9%) had no stenosis, 21 (29.2%) had stenosis between 20% and 30%, and 10 (13.9%) had stenosis greater than 30% (*p* = 0.03). A post hoc analysis confirmed that women without stenosis had a worse QoL compared to those with stenotic nodules (*p* = 0.045). The duration of therapy (whether more or less than 24 months) did not affect the results observed in these three groups of patients. No statistical associations were found between the duration of therapy or the characteristics of BE.

All these results affirm the hypothesis that the anatomical features of BE, particularly nodule size and stenosis, are critical determinants of patient outcomes, reinforcing the importance of individualized treatment strategies.

## 4. Discussion

The management of BE can be challenging for healthcare providers. If it does not cause severe obstruction to fecal transit, surgical intervention may not be the best option, as it is associated with a higher rate of ileostomy procedures and potential complications such as suture leakage, rectovaginal fistula formation, anastomosis stenosis, atonic bladder, and de novo bowel dysfunction, even when performed using nerve- and vessel-sparing techniques [7,21,22,23]. While surgical conservative approaches, such as discoid resection and rectal shaving, may result in fewer complications compared to segmental resection [24], intermediate and long-term bowel dysfunction, also known as Low Anterior Resection Syndrome (LARS), can still occur [25]. Additionally, rectal infiltration is often associated with parametrial involvement, often necessitating radical surgery with parametrectomy. Even with nerve-sparing approaches, such as the Negrar method routinely adopted at our institution, the prevalence of LARS remains significant [7,25,26,27].

Therefore, after the diagnosis of endometriosis, it is crucial to first evaluate whether surgery can be avoided or postponed, considering it only in cases of severe (sub)occlusive intestinal symptoms, ureteral stenosis with hydroureteronephrosis, the presence of large or suspicious adnexal masses, or contraindications or poor responses to medical therapy [8].

The current study analyzed VAS scores for pain and QoL using a validated questionnaire in approximately 600 patients with BE avoiding the surgical approach. Regardless of ongoing medical therapy, we found that these patients had a satisfactory mean QoL, with no significant intestinal symptoms related to the disease. Additionally, a minimal intensity of pain symptoms with a subsequent low impact on daily life was observed. This finding further confirms that the severity of symptoms and endometriosis staging are not necessarily correlated.

An early diagnosis is essential for both asymptomatic patients with BE, to prevent the progression of endometriosis through regular clinical follow-ups, and for highly symptomatic women who have only adenomyosis or superficial endometriosis without clear nodules of deep endometriosis. In our study, smaller nodules (less than 3 cm) and nodules not causing significant stenosis (less than 30%) were the most symptomatic. However, this finding may reflect a misinterpretation of the data, as it is more likely that larger, more painful nodules would have an indication for surgery (and thus be excluded from this study), while smaller ones are preferably managed conservatively with medical therapy, at least initially. Furthermore, the presence of bowel fixity and angulations associated with BE may exacerbate symptoms in patients with smaller nodules and those not causing significant stenosis. Ultimately, this finding may further confirm that many characteristics of BE nodules (size, degree of stenosis, number of locations) do not significantly impact QoL or symptom intensity, as previously suggested in another study [25].

On the other hand, the location of BE nodules appears to have clinical relevance. Specifically, low (rectal or rectosigmoid) nodules seem to be less tolerated compared to sigmoid nodules, as these patients exhibited worse results for VAS scores related to NMPP and dyschezia and experienced a more negative psychological impact on daily life (particularly in the EHP-5 subdomains of lack of control and powerlessness). This finding could be attributed to the anatomical spread of endometriosis: when the rectum is involved, it is very common to encounter the so-called “frozen pelvis” clinical presentation, characterized by a deep infiltration of the rectovaginal septum, pelvic ligaments, pelvic viscera (such as uterosacral ligaments, rectovaginal ligaments, lateral rectal ligaments, cardinal ligaments, uterus, vagina, etc.), and the posterior and lateral pelvic wall, located close to the visceral and somatic pelvic nerves [26]. This visceral and neural infiltration, or its compressive effect on the pelvic wall, is the main cause of the neurological pain reported by patients with endometriosis. In contrast, sigmoid BE may be isolated without complete involvement of the posterior compartment, potentially having a lesser impact on pelvic innervation and thus causing less pain.

A key finding of this study is the significant impact of hormonal therapy on QoL. The results indicate that patients receiving therapy, compared to those not undergoing hormonal treatment, demonstrate markedly better outcomes in terms of intestinal function at BENS, pain (EHP-5 pain subdomain, VAS scores for dysmenorrhea, dyschezia, dysuria, and NMPP), and psychological impact (EHP-5 control and powerlessness subdomains). Currently, several medications are available to manage BE, aiming to reduce circulating hormones and thereby induce a pseudo-menopausal or pseudo-pregnancy state, with significant improvements in patients’ QoL. Our study confirms that, in cases of BE, hormonal treatment is recommended regardless of nodule size or degree of stenosis, as previously suggested by other authors [28].

When therapy becomes ineffective over time, it is recommended to try at least one additional therapy modification before considering medical treatment failure. This could involve changing the class of hormones or the route of administration. However, hormones should not be regarded as a cure-all; while they may be effective in controlling pain, continuous long-term therapies are often associated with persistent side effects and the inability to achieve pregnancy, both of which can negatively impact QoL.

In this study, patients who were postmenopausal at follow-up after a previous diagnosis of BE had the best QoL, even surpassing that of reproductive-age patients under medical therapy. Given this finding, the potential side effects of treatment, and the fact that endometriosis is often associated with an earlier onset of menopause, it is reasonable to weigh the risks and benefits of medical therapy in patients nearing menopause. In this setting, hormone therapy should be considered only in cases of significant symptoms [8,28].

In definitive, this study highlights the viability of conservative management in women with bowel endometriosis, emphasizing that surgery may often be avoided, even in cases involving rectal nodules. Medical therapy, particularly hormonal treatments, significantly improves pain symptoms and QoL, offering a valid alternative to surgical intervention. These findings advance current understanding by demonstrating that conservative management can be equally effective in preserving QoL, even in anatomically complex cases such as rectal involvement.

### Study Strenghts and Limitations

One of the main strengths of this study is the large patient population, facilitated by the high volume of deep endometriosis cases treated annually at our hospital, which attracts patients from all over Italy. Over the years, this high volume of cases has significantly improved our accuracy in detecting endometriosis through both first- and second-level exams, enabling us to conduct a study on intestinal endometriosis without requiring histological confirmation, also according to the last 2022 ESHRE guidelines [12]. Additionally, to our knowledge, this is the first study to evaluate QoL and treatment compliance based on the characteristics of BE, providing clinicians with valuable insights for successfully managing the condition outside the operating room.

However, there are some limitations to this study. Retrospective data were used, and symptoms before treatment were collected from clinical notes and patient recall, which may affect the accuracy of symptom reporting. QoL was recorded only at the time of the phone call using standardized questionnaires (EHP-5 and BENS), resulting in a lack of longitudinal data to assess changes over time. The diagnosis of BE was made with the aid of the DCBE and TVS without histological confirmation, which may impact diagnostic accuracy. The major limitation of this study is that it does not consider very symptomatic patients who went directly to surgery or women who did not find relief with medical therapies and subsequently underwent surgery. Thus, we can infer that the population analyzed had an acceptable QoL, allowing them to avoid surgery. However, since the aim of this study was to evaluate whether patients with BE can achieve a satisfactory QoL with conservative management, rather than comparing outcomes between surgical and non-surgical patients, this limitation is less significant. Finally, this study was conducted at a single institution, which may limit the generalizability of the findings to other settings or populations.

## 5. Conclusions

Annually, between 13,000 and 15,000 patients are evaluated at the IRCCS “Sacro Cuore-Don Calabria” Hospital, with approximately 1500 (~10%) undergoing surgery. Although many patients are operated on each year—given that the IRCCS “Sacro Cuore-Don Calabria” is a dedicated center that manages the most severe cases from across Italy—the majority of women are treated conservatively. This conservative approach is also applied in cases of infiltrating BE, with the aim of avoiding or postponing bowel surgery. Women with BE who do not exhibit (sub)occlusive symptoms associated with a high degree of bowel stenosis do not necessarily require surgery, as they can maintain a good and lasting QoL regardless of the size, number, or location of nodules. The broader significance of this approach is the potential to reduce the frequency of high-risk surgeries, thus minimizing the associated complications and long-term morbidities of bowel surgeries, particularly in specialized centers where patients with severe forms of the disease are managed. Psychological support is also recommended, especially for patients undergoing long-term medical therapy, as chronic treatment can impact daily life.

Data from our study, confirming that the various characteristics of BE do not significantly impact QoL or symptom intensity, and that medical therapy may have a beneficial effect when treating patients with BE during their fertile years, support the hypothesis that BE can be managed conservatively in the majority of cases. This reinforces the notion that the “best surgery” for patients with deep endometriosis may often be the one that is never performed. By focusing on symptom management and enhancing QoL through non-invasive methods, clinicians can avoid overtreatment and instead provide individualized care based on the patient’s specific clinical needs and preferences.

Centers of care around the world that handle a high volume of surgical patients with endometriosis should recognize the responsibility to demonstrate that conservative treatment is a viable option for the majority of patients, even in cases involving the bowel. By advocating for evidence-based conservative management in high-volume centers, there is the potential to shift the paradigm from aggressive surgical intervention to more sustainable long-term care strategies. Treatment should be tailored to the patient, not solely to the disease, marking a shift from a lesion-oriented approach to a patient-oriented approach.

Future research should focus on refining conservative treatment strategies, understanding their long-term benefits and further elucidating the relationship between anatomical factors and symptom severity. Moreover, this study sets the foundation for developing evidence-based guidelines that prioritize non-invasive management in BE, potentially reducing the burden of surgical interventions across the field of endometriosis care. By prioritizing a patient-centered approach over a lesion-centered one, we can better tailor treatments to individual needs, improving both clinical outcomes and overall well-being.

## Figures and Tables

**Table 1:** Demographic characteristics of the study population (n = 580).

**Age (years; mean ± SD)**	40.64 ± 6.91
**BMI (kg/m^2^, mean ± SD)**	22.66 ± 4.28
**Timing follow-up (months mean ± SD)**	37.30 ± 19.75
**BE location (n, %)**	
Rectum	158 (27.2%)
Rectosigmoid junction	199 (34.3%)
Sigma	203 (35%)
Cecum/ileum/appendix	20 (3.5%)
**BE dimension (n, %)**	
<2 cm	215 (37.1%)
≥2 cm and <3 cm	212 (36.5%)
≥3 cm	153 (26.4%)
**Intestinal lumen stenosis (evaluated at DCBE; n, %)**	
No stenosis	293 (50.5%)
<30%	133 (22.9%)
≥30% and <50%	123 (21.2%)
≥50%	31 (5.4%)
**Number of BE nodules (n, %)**	
1	517 (89.1%)
2	62 (10.7%)
3	1 (0.2%)

BE: bowel endometriosis; BMI: body mass index; DCBE: double contrast bowel enema.

**Table 2:** Pain symptoms and quality of life in the study group assessed using the visual analogue scale (VAS), the bowel endometriosis syndrome (BENS) score, and the endometriosis health profile (EHP-5), which was divided into five subgroups (EHP 1 to 5) and also combined into a total score (EHP TOT).

Score (Mean ± SD)	All Patients (n = 580)	Patients in Reproductive Age Under Medical Therapy (n = 394)	Patients in Reproductive Age Without Medical Therapy (n = 147)	Menopausal Patients Without Medical Therapy (n = 39)
Dysmenorrhea, VAS	1.49 ± 2.72	0.41 ± 1.46	4.77 ± 3.02 *	0.13 ± 0.52
Dyschezia, VAS	2.2 ± 2.04	2.07 ± 1.86	2.83 ± 2.48 *	1.26 ± 1.02 *
Dyspareunia, VAS	2.91 ± 2.61	2.91 ± 2.6	3.01 ± 2.59	2.51 ± 2.77
Dysuria, VAS	1.23 ± 0.95	1.18 ± 0.85	1.39 ± 1.26 *	1.08 ± 0.48
NMPP, VAS	2.74 ± 2.23	2.63 ± 2.11	3.31 ± 2.52 *	1.72 ± 1.68 *
BENS SCORE	4.89 ± 5.28	4.62 ± 4.88	6.3 ± 6.28 *	2.38 ± 3.43 *
EHP pain (1)	7.19 ± 18.87	5.93 ± 17.19	12.16 ± 23.71 *	1.28 ± 8
EHP-2 lack control and powerless (2)	19.28 ± 30.15	18.62 ± 29.39	24.49 ± 33.28 *	6.41 ± 19.63 *
EHP-3 emotional (3)	27.9 ± 30.64	28.7 ± 30.64	27.05 ± 30.44	23.08 ± 21.61
EHP-4 social support (4)	28.51 ± 33.78	29.15 ± 34.07	31.16 ± 33.67	12.18 ± 26.82 *
EHP-5 self-image (5)	23.79 ± 30.41	25.64 ± 30.6	22.43 ± 30.47	10.26 ± 24.81 *
EHP TOTAL SCORE	105.42 ± 99.98	107.15 ± 96.5	115.99 ± 110.22	48.08 ± 74.20 *

BENS: Bowel endometriosis syndrome; EHP-5: Endometriosis health profile; VAS: visual analog scale; * = *p* < 0.005.

## Data Availability

The data presented in this study are available on request from the corresponding author due to the absence of an online dataset.
